# Differentiation of Parkinson’s disease tremor and essential tremor based on a novel hand posture

**DOI:** 10.1016/j.prdoa.2022.100146

**Published:** 2022-05-21

**Authors:** Sujitha Mahendran, Oliver Bichsel, Roger Gassert, Christian R. Baumann, Lukas L. Imbach, Daniel Waldvogel

**Affiliations:** a Department of Neurology, University Hospital Zurich, University of Zurich, Zurich Switzerland; b Clinical Neuroscience Centre, University Hospital Zurich, University of Zurich, Zurich, Switzerland; c Department of Neurosurgery, University Hospital Zurich, University of Zurich, Zurich, Switzerland; d Rehabilitation Engineering Laboratory, Department of Health Sciences and Technology, ETH Zurich, Zurich, Switzerland; e Swiss Epilepsy Center, Klinik Lengg, Zurich, Switzerland

**Keywords:** Parkinson's disease, Essential tremor, Tremor, Hand postures, Accelerometry

## Abstract

**Background:**

Tremor is one of the most common movement disorders but the correct diagnosis of tremor disorders, especially the differentiation between Parkinson’s disease tremor (PT) and essential tremor (ET) remains a challenge for clinicians.

**Method:**

We examined a novel hand position to distinguish PT from ET. We prospectively collected accelerometric tremor data in 14 ET patients and 14 PT patients with arms and hands fully stretched against arms stretched and hands relaxed, i. e. hanging down. The total acceleration from the three pairwise-perpendicular accelerometric axes during the 1-minute blocks of the two hand positions were computed and high-passed filtered at 2 Hz. The power spectral density during each hand position was calculated and summed up over the frequency domain.

**Results:**

Our results showed a significantly higher occurrence of tremor in the hands hanging down position in PT patients compared to ET patients (*p* = 0.0262). Moreover, in PT patients the tremor intensity significantly increased when transitioning from the stretched hand position to the hanging-down position (83 % of cohort) and vice versa in ET patients (75 % of cohort).

**Conclusion:**

In conclusion, the new hand posture can differentiate between PT and ET with high accuracy (sensitivity 83 %, specificity 75 % for PT) and may be a helpful tool in the clinical assessment of tremor.

## 1 Introduction

Patients presenting with tremor pose a challenging clinical scenario as an accurate diagnosis is crucial for the best medical therapy. Unfortunately, misdiagnoses are common and heavily impact clinical care [1]. Currently, clinical evaluation by a movement disorders specialist is the diagnostic gold standard for tremor syndromes [2], but has only moderate diagnostic accuracy when assessed against post-mortem histology [3]. The diagnostic accuracy of clinical diagnosis of Parkinson’s disease (PD) is estimated to be 80% amongst movement disorders experts and 74% amongst neurologists not specialized in movement disorders [4].

The most common tremor syndromes are Parkinson’s disease tremor (PT) and essential tremor (ET) [5], where the distinction is crucial because treatment and prognosis are substantially different.

The International Parkinson and Movement Disorder Society defines tremor as an involuntary, rhythmic oscillatory movement of a body part and proposed the classification of tremor syndromes based on two axes: clinical features and aetiology. One of the important clinical features is the activation condition [6]. The tremor in patients with PT is classically resting tremor [7], which occurs in a body part that is not voluntary activated and completely supported against gravity [6], [8]. Contrarily, ET characteristically is an action tremor occurring while voluntarily maintaining a position against gravity (postural tremor) or during any voluntary movement (kinetic tremor, intention tremor) [5], [6], whereas in late stages also rest tremor can be observed [9]. The suppression of rest tremor during movement onset and its reoccurrence after a latency is defined as re-emergent tremor [10], which is a relevant diagnostic feature to separate PT from ET [11].

Although nuclear imaging techniques exist, such as DaTSCAN [12], the overall accuracy of modern nuclear imaging techniques may not be different from that of clinical diagnosis established by a movement disorder specialist [13] and is only accessible in some specialised centres [14].

While there is general consensus that the patient’s arm should be relaxed and supported against gravity when examining resting tremor, there is no specific recommendation for best arm position [8]. Deuschl et al. recommended evaluating rest tremor while resting on a couch [5], others suggested lying down on a bed or arms hanging from the sides [15], moreover hands resting in his/her lap [16] and hands placed on the arm rest [17]. Wilken et al. investigated different hand positions to assess rest tremor in PD patients, where hands hanging down from the armrest showed the highest sensitivity and hands completely stretched on the armrest showed the greatest specificity for detecting rest tremor [8]. However, still there is no consensus on which arm position is the best to evaluate rest tremor [9], [8] and, interestingly, just few investigations regarding the distal hand position have been conducted [8].

Previously, we have used inertial measurement units for tremor classification [18] and prediction of treatment response [19]. A novel examination protocol using inertial measurement units is explored that captures rest tremor with high discriminatory accuracy. We hypothesised that stretched arms with hands hanging down is a favourable position to assess rest tremor. For this hand position, no voluntary muscle activation is required and the hands do not need to be placed on a surface. A combination of this novel posture with arms outstretched with hands stretched as well or arms outstretched with hands hanging could differentiate PT from ET.

## 2 Methods

### 2.1 Study cohort

In this monocentric observational study, a total of 14 PT (Table 1) and 14 ET patients (Table 2) were included between June 2017 and May 2018. All neurological patients with tremor were recruited from our outpatient movement disorders clinic or our inpatient wards at the Department of Neurology at the University Hospital Zurich after their written informed consent. We included male and female subjects aged between 18 and 90 years suffering from Parkinson’s disease or Essential Tremor, diagnosed by a movement disorder specialist based on clinical evaluation and treatment response. We excluded patients with significant concomitant disease (psychiatric or somatic) that would otherwise interfere with adherence to the study protocol. Study participation did not influence the medical management of any patient. All neurological examinations (including scores), demographics, medical history data, as well as the diagnosis of the tremor aetiology were performed by movement disorders specialists of the Department of Neurology at the University Hospital Zurich. Scores for PD patients included the third part of the Movement Disorder Society-Unified Parkinson’s Disease Rating Scale (MDS-UPDRS) [22], [23], ON/OFF values of the L-Dopa challenge test. Scores for the ET cohort included the ON/OFF values of the alcohol challenge (2–4 dl of 12.5 % vol. red wine) for the Washington Heights-Inwood Genetic Study of ET (WHIGET) Score [24]. Of our ET cohort, 10 could be classified as classical ET and 4 as ET-plus, none of these patients had a dystonic tremor. This study was approved by the local ethics committee (Kantonale Ethikkommission Zürich, BASEC-ID: 2018–00618), and all patients gave informed written consent prior to inclusion.

**Table 1:** Demographics and clinical patient characteristics of the PD patient cohort. The percentage in the ON/OFF column represents the improvement with respect to OFF. Patients who display an approximately equal degree of akinetic/rigid type and tremor-dominant type are listed as equivalent.

**ID**	**Age [y]**	**Gender**	**PD type**	**Disease duration [y]**	**MDS-UPDRS III ON/OFF**
1	71	M	tremor	2	24/37 (35 %)
2	75	F	tremor	15	62/85 (27 %)
3	70	M	tremor	2	10/23 (57 %)
4	66	F	tremor	5	12/29 (59 %)
5	44	M	tremor	5	8/33 (76 %)
6	41	M	tremor	4	17/24 (29 %)
7	76	M	tremor	12	32/49 (35 %)
8	55	M	tremor	2	28/43 (35 %)
9	60	F	equivalent	8	11/50 (78 %)
10	81	F	tremor	2	29/38 (24 %)
11	69	F	equivalent	8	46/51 (10 %)
12	62	F	equivalent	1	13/28 (54 %)
13	80	F	tremor	1	40/55 (27 %)
14	68	M	tremor	7	22/50 (56 %)

**Table 2:** Demographics and clinical patient characteristics of the ET patient cohort.

**ID**	**Age [y]**	**Gender**	**Disease duration [y]**	**WHIGET Score ON/OFF**
1	75	F	1	11/24
2	51	F	2	0/2
3	63	M	4	n.a./n.a.
4	75	F	4	8/25
5	63	F	1	4/10
6	52	M	1	n.a./7
7	81	M	20	13/15
8	80	M	n.a.	n.a./n.a.
9	69	F	5	n.a./27
10	71	F	11	n.a./28
11	62	F	30	n.a./10
12	77	F	2	n.a./13
13	67	M	2	13/23
14	46	F	21	7/16

### 2.2 Data collection

**Fig. 1:**
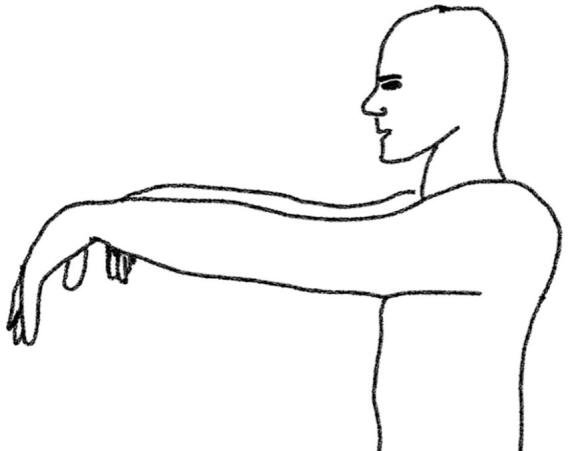
Hands hanging down position (HD).

**Fig. 2:**
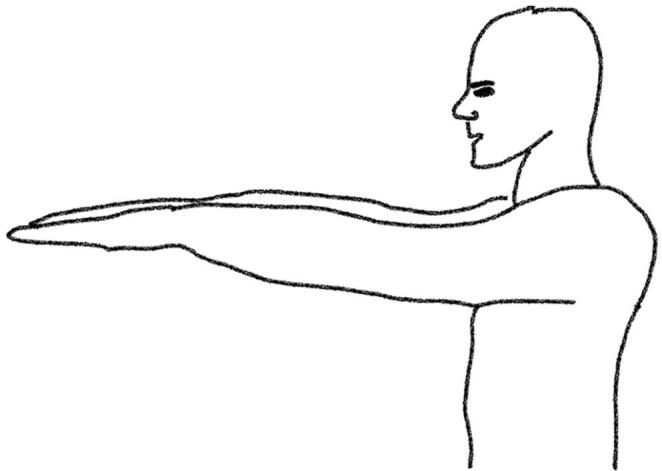
Hands completely stretched (CS).

Throughout the experiments, patients in OFF were videorecorded while sitting at a table, facing the camera with their hands clearly visible above the table. We investigated the ‘hands hanging down’ position (HD, Fig. 1) and the ‘hands completely stretched’ (CS, Fig. 2) during 1-minute blocks of shoulder anteversion to 90*^◦^*. For the purpose of precisely recording motion data, we used ReSense, a low-power 10-degrees-of-freedom inertial measurement unit (IMU) of only 15 g comprising a 3-axis accelerometer, a 3-axis gyroscope, a 3-axis magnetometer and a barometric pressure sensor [20]. This module recorded continuously at a sampling rate of 50 Hz. The IMUs (one per hand) were temporally synchronized via a base station prior to the measurement. The recorded data from the IMUs and the video were saved for data analysis.

### 2.3 Data analysis

The accelerometric and time data from the IMUs were imported into MATLAB (R2019b, the MathWorks Inc., Natick, Massachusetts, USA). A custom written program calculated the total (absolute) acceleration from the three pairwise perpendicular accelerometric axes using the formula:

$${\mathrm{acc}}_{\mathrm{tot}}=\sqrt{{\mathrm{acc}}_{x}^{2}+{\mathrm{acc}}_{y}^{2}+{\mathrm{acc}}_{z}^{2}}$$

The spectrogram function in MATLAB (window = 100, Hamming window, 50 % window overlap) was used in order to compute a short-time Fourier transform. Thanks to transitional movements between positions, this representation of the short-time Fourier transform allowed to visually recognize and segment the one-minute blocks for each hand position. All segments were high-pass filtered (3rd order Butterworth) at a frequency of 2 Hz and the first 5 and last 10 s of each block as well as the IMU data from the non-tremor-dominant hand discarded. The power spectral density (PSD) estimate was computed via Welch’s method, similar as in [21] (up to 8 segments with 50 % overlap, Hamming window). The total spectral power was calculated with the cumulative sum of elements, the power spectral densities for stretched vs. hanging positions were averaged over all PT patients and ET patients, retrospectively.

### 2.4 Statistical analysis

The alpha-level was set to 0.05. Wilcoxon rank sum test was used to test for differences in the tremor total spectral power between PT vs. ET during each hand position. Wilcoxon signed rank test was used to compare the tremor total spectral power between the two hand positions for each PT and ET.

## 3 Results

A total of 14PT patients (65.5 ± 12.25 years of age, 5.25 ± 4.25 years of disease duration; mean ± standard deviation) and 14 ET patients (66.5 ± 11 years of age, 8 ± 9.5 years of disease duration; mean ± standard deviation) were included in this study. Two patients from the PT group (IDs 9 and 12) and two patients from the ET group (IDs 12 and 13) had to be excluded due to either missing videorecording or (partly) missing IMU data.

**Fig. 3:**
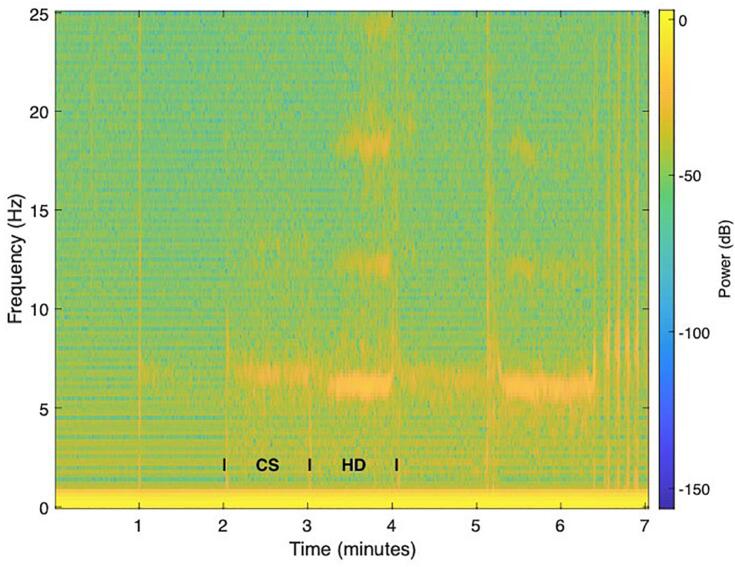
IMU total accelerometry spectrogram during the clinical tremor evaluation of a representative patient in our cohort. Transitions between 1-min blocks are seen as discrete power increases at certain time points (e. g. at 1 min, 2 min etc.). Here, the focus was on the ‘hands completely stretched (CS) and the ‘hands hanging down’ (HD). A main tremor frequency between ∼ 5–8 Hz can already be visually appreciated in the spectrogram, as well as harmonics between *t* = 3–4 min and *t* = 5–6. During the last minute, the patient was performing a target-to-nose test.

**Fig. 4:**
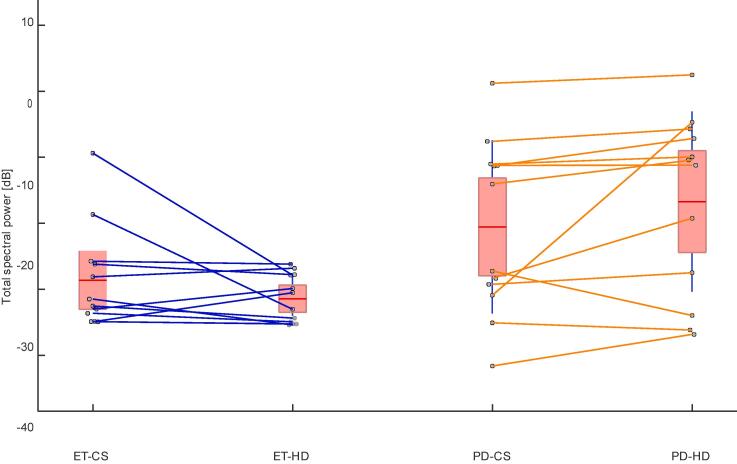
Comparing total tremor spectral power (mean: red horizontal line; 95 % confidence interval: red patched area; standard deviation: blue vertical line) during the ‘hands hanging down’ (HD) and ‘hands completely stretched’ (CS) positions in patients with Parkinson disease (PD, *n* = 12) and patients with essential tremor (ET, *n* = 12). Data from the same patient were connected by a line. Tremor increased significantly in patients with PD when transitioning from the CS to the HD hands position (Wilcoxon signed rank test, *p* = 0.0068). In contrast, tremor decreased in 75 % of ET patients during the transition from CS to the HD hands position. Moreover, tremor was significantly higher in PD as compared to ET during the HD hands position (Wilcoxon rank sum test, *p* = 0.0262). (For interpretation of the references to colour in this figure legend, the reader is referred to the web version of this article.)

**Fig. 5:**
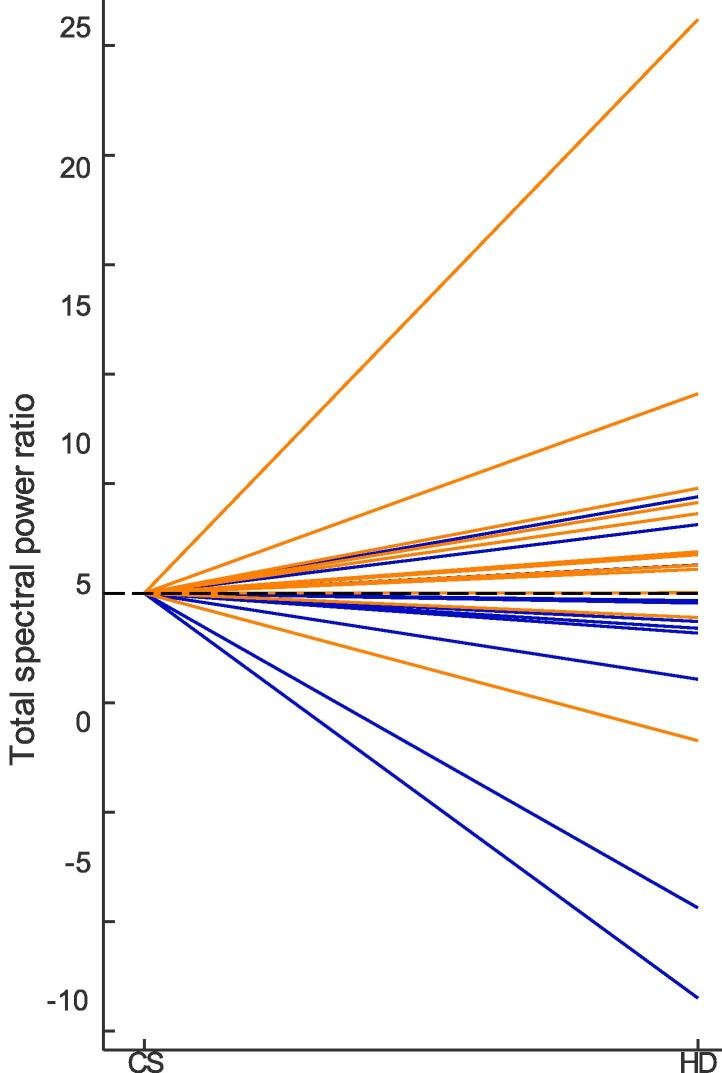
Change in the total spectral power for the transition between the ‘hands completely stretched’ (CS) to the ‘hands hanging down’ (HD) positions for patients with Parkinson disease (PD, orange lines) vs. patients with essential tremor (ET, blue lines). 77 % of patients that showed an increase in tremor from the CS to HD hands position had PD. 82 % of patients that showed a decrease in tremor from the CS to the HD hands position had ET. (For interpretation of the references to colour in this figure legend, the reader is referred to the web version of this article.)

After computing a spectrogram from the accelerometry data (Fig. 3), the total spectral power of tremor was calculated separately for the CS and HD hands position (Fig. 4). Tremor intensity was significantly higher in PT as compared to ET during the HD hands position (Wilcoxon rank sum test, *p* = 0.0262). Tremor increased significantly in patients with PT when transitioning from the CS to the HD hands position (Wilcoxon signed rank test, *p* = 0.0068). In contrast, tremor decreased in 75 % of ET patients during the transition from CS to the HD hands position. 77 % of patients who showed an increase in tremor from the CS to HD hands position had PD. 82 % of patients that showed a decrease in tremor from the CS to the HD hands position had ET (Fig. 5). An increased tremor during HD as compared to CS has a sensitivity of 83 % and specificity of 75 % for PT.

## 4 Discussion

Patients presenting with tremor depend on an accurate diagnosis for optimal treatment [1]. However, the current diagnostic standard, i. e. the clinical evaluation by a movement disorders specialist [2], has only moderate diagnostic accuracy resulting in a considerable number of misdiagnoses [3], [4]. By definition, rest tremor should be assessed in a position without voluntary activation and completely supported against gravity [8]. Here, a novel hand position was explored that can be used for detecting rest tremor and discriminate patients with ET and PT.

The findings of this study show an inverse tremor activity pattern for patients with PT vs. ET when transitioning between the two hand positions. Notably, there is a significant increase of tremor activity in patients with PT when transitioning from the CS to the HD position, which is in line with the clinical description of parkinsonian tremor being primarily a resting tremor and the decreased tremor activity during CS attributable to the delayed appearance of a postural tremor that is known as re-emergent tremor. Moreover, a high tremor activity during the HD position is indicative of PD, since tremor activity during the HD position is significantly higher in PD than ET. This reinforces our hypothesis that this new hand position HD is very much suitable for assessing rest tremor. Interestingly, only in HD higher harmonic oscillations were detected, which we described as dopamine-responsive pattern in our previous study. Lastly, the patients that had a decrease in tremor activity from CS to HD were in 82 % of the cases patients with ET, while the population with an increase in activity were in 77 % of the cases PD patients.

Given a small part of ET having tremor activity in HD, it remains open whether this is due to the fact that in this position only the hand and not the whole arm is supported against gravity.

This study is limited by the possibility of misdiagnoses of the true tremor aetiology, despite the extensive clinical workup, examination, history taking and follow up. Misdiagnosed tremor aetiologies would falsely reduce the herein reported ability of the IMUs to discriminate between ET and PT. Another limitation is that some patients only displayed low-amplitude total spectrum tremor power, sometimes even during both hand positions. These cases are not only clinically challenging to differentiate but also objectively using IMUs. Low tremor amplitudes are typically present at early stages of ET or PT, where the differentiation between the two tremor aetiologies is particularly difficult [22]. Patients presenting initially with parkinsonism and/or tremor characterised by a positive response to levodopa at the acute levodopa challenge have benefited from shorter delays until the final clinical diagnosis [23]. It remains to be seen whether the combined information of objective neurophysiological testing with current clinical evaluation increases the diagnostic accuracy. Unexpected patterns of tremor activation should prompt a repeated reflection of the leading diagnosis. Moreover, this study is limited by a rather small sample size (12 patients per cohort), and thus a replication within larger cohorts is required.

In conclusion, the novel hand position HD enables the detection of rest tremor and helps in the differentiation between PT and ET, especially when comparing tremor activity between different hand positions and during the transitions. We therefore consider it a useful tool for the assessment of tremor patients and would like to encourage its use in daily clinical practice.

## Funding

This research did not receive any specific grant from funding agencies in the public, commercial, or not-for-profit sectors.

## 6 Contribution

Authors have made substantial contributions to the conception and design of the study (DW, LLI), acquisition of data (SM, OB, RG), analysis and interpretation of data (SM, OB, CB, LLI, DW), drafting the article (SM, OB), revising it (all authors) and gave final approval of the submitted version (all authors).

## Declaration of Competing Interest

The authors declare that they have no known competing financial interests or personal relationships that could have appeared to influence the work reported in this paper.
